# Supplementary material for: Continuous glucose monitoring in obese pregnant women with no hyperglycemia on glucose tolerance test
Source: PLoS One. 2021 Jun 10;16(6):e0253047. doi: 10.1371/journal.pone.0253047 (PMC8191902; doi:10.1371/journal.pone.0253047)
Supplement: Attachment — Submitted filename: PONE-D-20-32574_reviewer.pdf [file pone.0253047.s002.pdf]

PLOS ONE

Continuous glucose monitoring in obese pregnant women with no hyperglycemia on glucose tolerance test

--Manuscript Draft--

**Manuscript Number:**	PONE-D-20-32574
**Article Type:**	Research Article
**Full Title:**	Continuous glucose monitoring in obese pregnant women with no hyperglycemia on glucose tolerance test
**Short Title:**	Continuous glucose monitoring in obese pregnant women
**Corresponding Author:**	Daiane de Oliveira de Oliveira Pereira Vergani, MS<br>University of Caxias do Sul<br>Caxias do sul, Rio Grande do Sul BRAZIL
**Keywords:**	obesity; pregnancy, high-risk; hyperglycemia
**Abstract:**	Objective: The objective of the present study was to compare 24-hour glycemic levels between obese pregnant women with normal glucose tolerance and non-obese pregnant women. Methods: In the present observational, longitudinal study, continuous glucose monitoring was performed in obese pregnant women with normal oral glucose tolerance test with 75 g of glucose between the 24 th and the 28 th gestational weeks. The control group (CG) consisted of pregnant women with normal weight who were selected by matching the maternal age and parity with the same characteristics of the obese group (OG). Glucose measurements were obtained during 72 hours. Results: Both the groups were balanced in terms of baseline characteristics (age: 33.5 [28.7–36.0] vs. 32.0 [26.0–34.5] years, p=0.5 and length of pregnancy: 25.0 [24.0–25.0] vs. 25.5 [24.0–28.0] weeks, p=0.6 in the CG and in the OG, respectively). Pre-breakfast glycemic levels were 77.77 ± 10.55 mg/dL in the CG and 82.02 ± 11.06 mg/dL in the OG (p<0.01). Glycemic levels at 2 hours after breakfast were 87.31 ± 13.10 mg/dL in the CG and 93.48 ± 18.74 mg/dL in the OG (p<0.001). Daytime blood glucose levels were 87.6 ± 15.4 vs. 93.1 ± 18.3 mg/dL (p<0.001) and nighttime blood glucose levels were 79.3 ± 15.8 vs. 84.7 ± 16.3 mg/dL (p<0.001) in the CG and in the OG, respectively. The 24-hour, daytime, and nighttime values of the area under the curve were higher in the OG when compared with the CG (85.1 ± 0.16 vs. 87.9 ± 0.12, 65.6 ± 0.14 vs. 67.5 ± 0.10, 19.5 ± 0.07 vs. 20.4 ± 0.05, respectively; p<0.001). Conclusion: The results of the present study showed that obesity in pregnancy was associated with higher glycemic levels even in the presence of normal findings on glucose tolerance test.
**Order of Authors:**	Rosa Maria Rahmi
	Priscila de Oliveira
	Luciano Selistre
	Paulo Cury Rezende
	Gabriela Neuvald Pezzella
	Pâmela Antoniazzi dos Santos
	Daiane de Oliveira de Oliveira Pereira Vergani, MS
	Sônia Regina Cabral Madi
	José Mauro Madi
**Additional Information:**	
**Question**	**Response**
**Financial Disclosure**<br><br>Enter a financial disclosure statement that	The authors received no specific funding for this work.

describes the sources of funding for the work included in this submission. Review the submission guidelines for detailed requirements. View published research articles from *PLOS ONE* for specific examples.<br><br>This statement is required for submission and **will appear in the published article** if the submission is accepted. Please make sure it is accurate.<br><br>**Unfunded studies**<br>Enter: *The author(s) received no specific funding for this work.*<br><br>**Funded studies**<br>Enter a statement with the following details:<br>• Initials of the authors who received each award<br>• Grant numbers awarded to each author<br>• The full name of each funder<br>• URL of each funder website<br>• Did the sponsors or funders play any role in the study design, data collection and analysis, decision to publish, or preparation of the manuscript?<br>• **NO** - Include this sentence at the end of your statement: *The funders had no role in study design, data collection and analysis, decision to publish, or preparation of the manuscript.*<br>• **YES** - Specify the role(s) played.<br><br>* typeset	
**Competing Interests**<br><br>Use the instructions below to enter a competing interest statement for this submission. On behalf of all authors, disclose any competing interests that could be perceived to bias this work—acknowledging all financial support and any other relevant financial or non-financial competing interests.<br><br>This statement **will appear in the published article** if the submission is accepted. Please make sure it is accurate. View published research articles from *PLOS ONE* for specific examples.	The authors have declared that no competing interests exist.

**NO authors have competing interests**<br><br>Enter: *The authors have declared that no competing interests exist.*<br><br>**Authors with competing interests**<br><br>Enter competing interest details beginning with this statement:<br><br>*I have read the journal's policy and the authors of this manuscript have the following competing interests: [insert competing interests here]*<br><br>* typeset	
**Ethics Statement**<br><br>Enter an ethics statement for this submission. This statement is required if the study involved:<br><br>• Human participants<br>• Human specimens or tissue<br>• Vertebrate animals or cephalopods<br>• Vertebrate embryos or tissues<br>• Field research<br><br>Write "N/A" if the submission does not require an ethics statement.<br><br>General guidance is provided below. Consult the submission guidelines for detailed instructions. **Make sure that all information entered here is included in the Methods section of the manuscript.**	Institution: Universidade de Caxias do Sul-RS-Brazil<br>Approval number: 73901317.7.0000.5341<br>Form of consent obtained: The participants signed the informed consent document before taking<br>part in study procedures.

Powered by Editorial Manager® and ProduXion Manager® from Aries Systems Corporation

**Format for specific study types**<br><br>**Human Subject Research (involving human participants and/or tissue)**<br>• Give the name of the institutional review board or ethics committee that approved the study<br>• Include the approval number and/or a statement indicating approval of this research<br>• Indicate the form of consent obtained (written/oral) or the reason that consent was not obtained (e.g. the data were analyzed anonymously)<br><br>**Animal Research (involving vertebrate animals, embryos or tissues)**<br>• Provide the name of the Institutional Animal Care and Use Committee (IACUC) or other relevant ethics board that reviewed the study protocol, and indicate whether they approved this research or granted a formal waiver of ethical approval<br>• Include an approval number if one was obtained<br>• If the study involved *non-human primates*, add *additional details* about animal welfare and steps taken to ameliorate suffering<br>• If anesthesia, euthanasia, or any kind of animal sacrifice is part of the study, include briefly which substances and/or methods were applied<br><br>**Field Research**<br><br>Include the following details if this study involves the collection of plant, animal, or other materials from a natural setting:<br>• Field permit number<br>• Name of the institution or relevant body that granted permission	
**Data Availability**<br><br>Authors are required to make all data underlying the findings described fully available, without restriction, and from the time of publication. PLOS allows rare exceptions to address legal and ethical concerns. See the PLOS Data Policy and FAQ for detailed information.	Yes - all data are fully available without restriction

A Data Availability Statement describing where the data can be found is required at submission. Your answers to this question constitute the Data Availability Statement and **will be published in the article**, if accepted.<br><br>**Important:** Stating 'data available on request from the author' is not sufficient. If your data are only available upon request, select 'No' for the first question and explain your exceptional situation in the text box.<br><br>Do the authors confirm that all data underlying the findings described in their manuscript are fully available without restriction?	
**Describe where the data may be found in full sentences. If you are copying our sample text, replace any instances of XXX with the appropriate details.**<br><br>• If the data are **held or will be held in a public repository**, include URLs, accession numbers or DOIs. If this information will only be available after acceptance, indicate this by ticking the box below. For example: *All XXX files are available from the XXX database (accession number(s) XXX, XXX.).*<br>• If the data are all contained **within the manuscript and/or Supporting Information files**, enter the following: *All relevant data are within the manuscript and its Supporting Information files.*<br>• If neither of these applies but you are able to provide **details of access elsewhere**, with or without limitations, please do so. For example:<br><br>*Data cannot be shared publicly because of [XXX]. Data are available from the XXX Institutional Data Access / Ethics Committee (contact via XXX) for researchers who meet the criteria for access to confidential data.*<br><br>*The data underlying the results presented in the study are available from (include the name of the third party*	No.

and contact information or URL).<br>• This text is appropriate if the data are owned by a third party and authors do not have permission to share the data.<br><br>* typeset	
Additional data availability information:	

Powered by Editorial Manager® and ProduXion Manager® from Aries Systems Corporation

Manuscript
Click here to access/download;Manuscript;renamed_d4126.docx

Original article

Full title:

Continuous glucose monitoring in obese pregnant women with no hyperglycemia on glucose tolerance test

Short title:

Continuous glucose monitoring in obese pregnant women

Rosa Maria Rahmi[1*], Priscila de Oliveira[2¶], Luciano Selistre[3&], Paulo Cury Rezende [4&], Gabriela Neuvald Pezzella[5&], Pâmela Antoniazzi dos Santos[6&], Daiane de Oliveira Pereira Vergani[7&], Sônia Regina Cabral Madi[8&], José Mauro Madi[8¶]

[1] *Department of Endocrinology, General Hospital of Caxias do Sul, Caxias do Sul University, Caxias do Sul, RS, Brazil*

[2] *Master degree student in Health Sciences, Caxias do Sul University, Caxias do Sul, RS, Brazil.*

[3] *Department of Nephrology, General Hospital of Caxias do Sul, Caxias do Sul, RS, Brazil*

[4] *Department of Atherosclerosis, Heart Institute of the University of São Paulo Medical School, São Paulo, SP, Brazil.*

[5] *Institutional Scientific Initiation Scholarship Program of Caxias do Sul University, Caxias do Sul, RS, Brazil*

[6] *Department of Nutrition, Caxias do Sul University, Caxias do Sul, RS, Brazil.*

[7] *Department of Nursing, Caxias do Sul University, Caxias do Sul, RS, Brazil.*

[8] *Department of Obstetrics and Gynecology, General Hospital of Caxias do Sul, Caxias do Sul University, Caxias do Sul, RS, Brazil*

* *Corresponding author:*

Rosa Maria Rahmi

E-mail: rmrgarcia@ucs.br (RMR)

Telephone: +55 54 99931-3441 | +55 54 3218-2100

¶ These authors contributed equally to this work.

& These authors also contributed equally to this work.

ORCID:

Rosa Maria Rahmi Orcid ID: https://orcid.org/0000-0003-1547-6282

Priscila de Oliveira Orcid ID https://orcid.org/0000-0002-2113-143

Luciano Selistre Orcid ID https://orcid.org/0000-0002-0152-0636

Paulo Cury Rezende Orcid ID https://orcid.org/0000-0001-8625-1537

Gabriela Neuvald Pezzella Orcid ID https://orcid.org/0000-0001-8989-8176

Pâmela Antoniazzi dos Santos Orcid ID: https://orcid.org/0000-0002-1197-8739

Daiane de Oliveira Pereira Vergani Orcid ID https://orcid.org/0000-0003-2510-1740

Sônia Regina Cabral Madi Orcid ID https://orcid.org/0000-0002-7039–8486

José Mauro Madi Orcid ID https://orcid.org/0000-0002-2345-4713

Abstract

Objective: The objective of the present study was to compare 24-hour glycemic levels between obese pregnant women with normal glucose tolerance and non-obese pregnant women. **Methods:** In the present observational, longitudinal study, continuous glucose monitoring was performed in obese pregnant women with normal oral glucose tolerance test with 75 g of glucose between the 24th and the 28th gestational weeks. The control group (CG) consisted of pregnant women with normal weight who were selected by matching the maternal age and parity with the same characteristics of the obese group (OG). Glucose measurements were obtained during 72 hours.**Results:** Both the groups were balanced in terms of baseline characteristics (age: 33.5 [28.7–36.0] vs. 32.0 [26.0–34.5] years, p=0.5 and length of pregnancy: 25.0 [24.0–25.0] vs. 25.5 [24.0–28.0] weeks, p=0.6 in the CG and in the OG, respectively). Pre-breakfast glycemic levels were 77.77 ± 10.55 mg/dL in the CG and 82.02 ± 11.06 mg/dL in the OG (p<0.01). Glycemic levels at 2 hours after breakfast were 87.31 ± 13.10 mg/dL in the CG and 93.48 ± 18.74 mg/dL in the OG (p<0.001). Daytime blood glucose levels were 87.6 ± 15.4 vs. 93.1 ± 18.3 mg/dL (p<0.001) and nighttime blood glucose levels were 79.3 ± 15.8 vs. 84.7 ± 16.3 mg/dL (p<0.001) in the CG and in the OG, respectively. The 24-hour, daytime, and nighttime values of the area under the curve were higher in the OG when compared with the CG (85.1 ± 0.16 vs. 87.9 ± 0.12, 65.6 ± 0.14 vs. 67.5 ± 0.10, 19.5 ± 0.07 vs. 20.4 ± 0.05, respectively; p<0.001).**Conclusion:** The results of the present study showed that obesity in pregnancy was associated with higher glycemic levels even in the presence of normal findings on glucose tolerance test.

Keywords: obesity; pregnancy, high-risk; hyperglycemia

Introduction

In the past few decades, the prevalence of obesity has increased, reaching the proportion of a global epidemic. In 2016, the World Health Organization (WHO) estimated that approximately 650 million adults were obese, representing approximately 13% of the world's adult population. Obesity affects all age groups and both sexes irrespective of the income levels [1]. Concomitant with the global increase in obesity, the number of obese pregnant women has also increased [2].

The association of obesity with pregnancy has been an important public health problem and a major challenge for the professional team responsible for assisting this population. Maternal obesity is associated with adverse pregnancy and perinatal outcomes and long-term complications related to maternal and fetal health [3].

Current evidence supports the strong association between obesity and gestational diabetes mellitus (GDM) [4,5]. Excess fat tissue releases increased amounts of unesterified fatty acids, glycerol, hormones, pro-inflammatory cytokines, and other factors that participate in the development of insulin resistance (IR). IR and dysfunctional beta-pancreatic cells are the main factors causing hyperglycemia [6,7]. In this context, maternal obesity causes imbalance in glycemic homeostasis during pregnancy, resulting in an increased risk of GDM [8].

Screening and diagnosis of GDM has improved in recent decades. However, there is still a lack of universally accepted consensus [9-11]. In 2010, the International Association of Diabetes in Pregnancy Study Group (IADPSG) [12] updated the diagnostic criteria based on the results of an important study, namely the Hyperglycemia and Adverse Pregnancy Outcomes (HAPO) study (13). These criteria were widely accepted by national and international organizations.

The HAPO study suggested a strong and continuous relationship between maternal blood glucose and adverse outcomes [13]. The study proposed a lower glycemic threshold to detect GDM compared to other international guidelines [9,14-16].

GDM is mainly diagnosed using the oral glucose tolerance test (OGTT), which is based on a limited number of plasma glucose level readings after glucose overload [16].

After diagnosis, GDM needs to be treated by a multidisciplinary team. Glycemic control supervised by glycemic self-monitoring at specific time points (especially preprandial and postprandial readings) is crucial to reduce the risk of adverse maternal and fetal outcomes [17].

During pregnancy, the proposed range of glycemic levels to manage hyperglycemia is more limited. This rigor is believed to positively influence the adverse perinatal outcomes. However, such monitoring is based on a limited number of analyses within 24 hours and long periods between meals are not monitored. Maternal blood glucose has a dynamic variation within 24 hours and is influenced by numerous factors such as insulin sensitivity, diet, lifestyle, stress, sleep, and others [18,19].

Currently, with technological developments in continuous glucose monitoring (CGM), it is possible to assess daily glycemic fluctuations with greater accuracy. Several studies have been designed to allow better understanding of the effect of hyperglycemia on the temporal behavior of glycemic levels in pregnancy [20-23]. However, very few studies have analyzed the continuous evolution of glycemic levels during the period in pregnancy without glucose intolerance [24-26]. Obese women with presumably normal glucose tolerance may experience adverse perinatal complications similar to those observed in women with GDM [4,27].

Thus, the present study was designed to continuously assess the glycemic levels of obese pregnant women without glucose intolerance according to the criteria proposed by the IADPSG (step one) and to compare them with glycemic levels of non-obese pregnant women (step two).

Materials and methods

The present prospective, observational, longitudinal study involving pregnant women was followed up by the Obstetrics and Gynecology Service of the General Hospital of the University of Caxias do Sul, RS, Brazil. The study was conducted from June 2018 to July 2019. We consecutively recruited pregnant women undergoing OGTT with 75 g of glucose between the 24th and the 28th gestational weeks. We included women with fasting glycemic levels below 92 mg/dL (5.1 mmol/L), 1-hour glycemic levels below 180 mg/dL (10.0 mmol/L), and 2-hour glycemic levels below 153 mg/dL (8.5 mmol/L). Pregnant women with pre-gestational obesity (body mass index [BMI] range: 30–40 kg/m^2) from the high-risk pregnancy clinic were included in the obese group (OG). Pregnant women from the low-risk prenatal clinic with normal pre-pregnancy weight (BMI range: 18.5–24.9 kg/m^2) were included in the control group (CG). The groups were matched (1:1) by maternal age, parity, and length of pregnancy.

Pregnant women aged 18 to 35 years and with gestational age between 24 to 32 weeks were included. The exclusion criteria were multiple pregnancies; fetal malformation; pregnant women with uncontrolled chronic diseases; smoking; alcoholism; and use of corticosteroids, beta-blockers, or hyperglycemic drugs.

Pregnant women were continuously monitored by the prenatal care team without any interference from the researchers.

The following data were collected from the medical records immediately after OGTT: age, pregestational BMI, parity, weight gain during pregnancy, gestational age at the time of OGTT, OGTT results (fasting, at 1 hour after overload, and at 2 hours after overload), family history of cardiovascular disease, and family history of diabetes. Pregestational BMI was calculated according to the WHO standards and expressed as weight (kg)/height $(m)^2$. Maternal weight gain during pregnancy was calculated by subtracting the body weight at the time of OGTT from the pre-pregnancy weight.

Continuous glucose monitoring

A CGM system iPro™2 Professional CGM, by Medtronic Principal Executive Office 20 Lower Hatch Street Dublin 2, Ireland), was used to measure interstitial glucose concentrations over a period of 24 hours for 3 consecutive days. The sensors were inserted in the subcutaneous tissue in the lower abdomen on the right or the left side. The sensors were connected to the transmitters attached to the skin. The sensor recorded approximately 288 blood glucose level readings in each pregnant woman over 24 hours. After 72 hours, the data were stored in a database. The monitors were calibrated by inserting capillary blood glucose level measured three times a day (preprandial measurements) using the Accu-Chek Active® device (Roche, Basel, Switzerland). Concomitantly, the women were requested to record the time at the start of the main meals and the time at the start of physical exercise.

The study was approved by the Ethics and Human Resources Committee of the University of Caxias do Sul (opinion No. 2,273,140). It was conducted according to the ethical principles of the Declaration of Helsinki. All participants signed an Informed Consent Form.

Statistical analysis

The data were expressed as mean ± standard deviation, median [interquartile range], and percentage. Exploratory analysis of the descriptive data was performed using Student's t-test, Wilcoxon-Mann-Whitney test, and Pearson's chi-squared test.

Since blood glucose concentrations of nestlings from the same brood are not independent, the glucose concentrations were analyzed using mixed linear models with brood identity included as a random controlling factor. In the first step, the glucose levels were modeled according to a linear mixed model with random intercept to quantify the effect of the group (obese or non-obese). The mean values of the two groups were compared using t-test in the linear mixed model. In the second step, two models were built: a first model that included variables "group" and "time" and a second model that included an interaction between the variables "group" and "time." The second model allowed quantification of the change in the effect of the group type according to time. Analysis of variance was used to compare the two nested models and to determine the statistical significance of the interaction. The models were adjusted by the restricted maximum likelihood method using the LME function of the NLME package. Tukey's post hoc test was used for multiple comparisons.

The analyses were performed using R for Windows, version 3.1.1 (R-Cran project, http://cran.r-project.org/, The R foundation, Vienna, Austria). Nominal p-values <0.05 were considered statistically significant.

Results and discussion

Altogether, 20 pregnant women were included and evaluated in this study. The baseline characteristics of the population in the OG (n=10) and in the CG (n=10) are described in Table 1. The median maternal age was 33.5 [28.7–36.0] years in the CG and 32.0 [26.0–34.5]

years in the OG (p=0.5). The pregestational BMI (kg/m^2) was 22.1 [21.7–23.8] in the CG and 39.9 [35.8–41.9] in the OG (p<0.001). Maternal weight gain until the day of OGTT tended to be greater in the OG (8.0 [5.5–10.7] kg) than in the CG (2.6 [0.00–8.6] kg) (p=0.09).

The analysis of OGTT results revealed that the fasting glycemic levels tended to be higher in the OG (75.5 [72.0–79.7] mg/dL) than in the CG (81.5 [74.2–87.0] mg/dL) (p=0.08). Blood glucose levels at 1 and 2 hours after glucose overload showed no significant differences between the groups. Moreover, no statistically significant difference was observed in parity and in family history of cardiovascular disease and diabetes between the groups (Table 1).

The CGM data of pregnant women from both the groups are presented in Table 2. A significant difference was observed in blood glucose levels before (77.77 mg/dl ± 10.55 vs. 82.02 ± 11.06, p<0.01) and 2 hours after breakfast (87.31 mg/dl ± 13.10 vs. 93.48 ± 18.74, p<0.001) between the CG and the OG. No significant difference was observed in the values within 1 hour after breakfast. No significant differences were observed in glucose levels before and after lunch and dinner between the groups.

Additionally, blood glucose levels during the day (between 6:00 am and 12:00 pm) were significantly higher in the OG compared to those in the CG (93.08 mg/dl ± 18.30 vs. 87.58 ± 15.40, p<0.001). Similarly, blood glucose levels at night (between 12:00 pm and 6:00 am) were significantly higher in the OG compared to those in the CG (84.73 mg/dl ± 16.31 vs. 79.35 ± 15.76, p<0.001)

The areas under the curve (AUCs) for blood glucose levels during the day and at night were 67.47 mg/dl ± 0.105 and 20.42 ± 0.05, respectively in the OG and 65.56 mg/dl ± 0.144 and 19.53 ± 0.072, respectively in the CG (p<0.001) (Table 2). The 24-hour AUC for blood glucose levels was 85.08 mg/dl ± 0.161 in the OG and 87.89 ± 0.116 in the CG (p<0.001) (Table 2, Figure 1).

Table 3 shows the isolated effect of obesity on longitudinal blood glucose variation. This effect was significant at night (78.10 mg/dl [95% confidence interval: 72.61–83.60] in the CG vs. 82.78 mg/dl [95% confidence interval: 78.60–86.96] in the OG, $p<0.001$).

The present study clearly showed a difference in temporal evolution of glycemic levels between obese and non-obese pregnant women without hyperglycemia according to the IADPSG criteria [12]. The national protocol in Brazil suggests that GDM screening should be performed using OGTT with 75 g of glucose between the 24th and the 28th gestational weeks in pregnant women with no previous glycemic changes. GDM is diagnosed when the following levels were reached or exceeded: fasting glucose level of 92 mg/dl, 1-hour level of 180 mg/dL, and 2-hour level of 153 mg/dL [16].

In the studied population, the analysis of blood glucose levels at fasting, at 1 hour, and at 2 hours after 75 g glucose overload confirmed that none of the pregnant women met or exceeded these criteria. However, fasting glycemic levels in the OG tended to be higher than those in the CG ($p=0.08$) at the time of screening.

None of the pregnant women in the study exhibited evidence of hyperglycemia. Therefore, they were routinely monitored without strict blood glucose level control until the end of pregnancy. No intervention was performed by the researchers. The objective of this study was to assess blood glucose levels without changing the routine in a population at high risk for metabolic diseases.

The obese pregnant women in the present study were referred to a reference center for high-risk pregnancies at the General Hospital of Caxias do Sul. There has been a significant increase in the number of women with severe obesity in recent years due to the global obesity epidemic that also affects women of reproductive age [2]. According to the study by Kim et al., the rate of GDM in a population with severe obesity (35–64.9 kg/m^2) was 11.5% and the

relative risk of GDM was 5.0 (95% confidence interval: 3.6–6.9) even after adjustment for maternal age, race/ethnicity, parity, and marital status [8]. In addition to pregestational BMI, weight gain during pregnancy may also be associated with an increased risk for GDM [28,29].

In the present study population, weight gain during the study period was higher in the OG (median: 8.00 kg) when compared with that in the CG (median: 2.65 kg), which is an additional factor for increased risk of hyperglycemia. Despite the high pregestational BMI and the greater weight gain in obese pregnant women, GDM was not detected at the time of screening. Thus, there is a possibility of dysglycemia in later stages of pregnancy in risk groups with a negative GDM test. Gomes et al. showed that among 448 obese pregnant women with a negative GDM test, 30.1% (n=135) exhibited dysglycemia at the end of the third trimester, as assessed by increased hemoglobin A1c levels [30]. A secondary analysis of the HAPO study in a population of 23,316 pregnant women showed that 2,247 (9.6%) women were obese without a diagnosis of hyperglycemia and this condition showed an independent association with fetal hyperinsulinemia, growth, and adiposity, similar to the outcomes observed in GDM [4]. This subject continues being discussed due to the scarce literature on the effects of late glycemic changes and maternal lipid profile [31,32] on perinatal outcomes.

Blood glucose levels at specific time points (2 hours before and 2 hours after breakfast) were significantly higher in the OG. However, the levels did not exceed the recommended limits for these time points (<95 and <120 mg/dl, respectively) [17]. These are the recommended time points to monitor pregnant women with hyperglycemia. At these time points, blood glucose levels remained within the presumably normal range in both the groups. Harmon reported significant differences in glycemic levels at 1 and 2 hours after meals [26]. Stratified

analysis by pregestational maternal weight conducted by Yogev et al. showed that preprandial, 1-hour postprandial, and 2-hour postprandial glycemic levels were significantly higher in obese pregnant women [25].

A detailed analysis of blood glucose samples repeated for 72 hours showed higher fluctuation in obese pregnant women than in non-obese pregnant women (assessed by the AUC). Similar behavior was observed when the analysis was divided into two periods (day and night). In addition, obesity was associated with a higher mean blood glucose at night. These data suggest that fetuses of the women from the OG could potentially be exposed to a blood glucose pattern that is higher than normal. These findings are consistent with the findings of Harmon et al. [26] who evaluated groups of pregnant women without hyperglycemia with and without dietary interference and reported that the AUC was always higher in obese pregnant women regardless of dietary control. In the present study, the OG included pregnant women with more severe obesity (median BMI: 39.95) and the criteria for excluding glucose intolerance in the population were different. However, Yogev et al. [25] showed that obese women exhibited significantly lower mean glucose levels at night compared to non-obese women.

Differences in glycemic homeostasis between obese and non-obese pregnant women were didactically presented by analyzing temporal blood glucose variations over long periods, which is possible only with the CGM systems. Despite the few studies available in the literature, the following questions should be discussed. 1) Should the glycemic targets for obese pregnant women be individualized? 2) Could the nocturnal glycemic changes be related to increased fetal fat and/or macrosomia in obese women without GDM?

Increasing maternal obesity rates have challenged researchers to characterize the metabolic profile of this population in a better way. Glycemic control is not adequately addressed during

the follow-up in most of the obese pregnant women without GDM. On the other hand, glucose self-monitoring has limitations, as it does not include the night period. The present study suggests the need for more evidence on glycemic targets in obese women during pregnancy. The sample size in the present study did not allow correlations with perinatal outcomes. However, the use of statistical modeling and the strict composition of the two groups clearly showed distinct behaviors in dynamic changes in blood glucose levels over long periods.

Conclusion

In conclusion, the present study demonstrated that continuously assessed blood glucose levels were higher in obese pregnant women without GDM than in non-obese pregnant women and this effect was more evident at night. Additional studies correlating these changes with fetal outcomes might contribute to a more personalized care for this population.

Acknowledgements

The authors are grateful to Hospital Geral de Caxias do Sul for receive this search.

References

1. World Health Organization. Obesity and overweight [Internet]. World Health Organization; Geneva: 2020. [cited 2020 Jul 24]. Available from: https://www.who.int/news-room/fact-sheets/detail/obesity-and-overweight

2. Poston L, Caleyachetty R, Cnattingius S, Corvalán C, Uauy R, Herring S, et al. Preconceptional and maternal obesity: epidemiology and health consequences. Lancet Diabetes Endocrinol. 2016;4(12):1025–1036. doi: 10.1016/S2213-8587(16)30217-0.

3. Catalano PM, Shankar K. Obesity and pregnancy: mechanisms of short term and long term adverse consequences for mother and child. BMJ. 2017;356:j1.4. doi.org/10.1136/bmj.j1

4 Catalano PM, McIntyre HD, Cruickshank JK, McCance DR, Dyer AR, Metzger BE,et al. The hyperglycemia and adverse pregnancy outcome study: associations of GDM and obesity with pregnancy outcomes. Diabetes Care. 2012;35(4):780–786. doi.org/10.2337/dc11-1790.

5. Plows JF, Stanley JL, Baker PN, Reynolds CM,Vickers MH . The Pathophysiology of Gestational Diabetes Mellitus. Int J Mol Sci. 2018;19(11):3342. doi: 10.3390/ijms19113342

6. Saltiel AR, Olefsky JM. Inflammatory mechanisms linking obesity and metabolic disease. J Clin Invest. 2017;127(1):1–4. doi.org/10.1172/JCI92035.

7. Pantham P, Aye IL, Powell TL. Inflammation in maternal obesity and gestational diabetes mellitus. Placenta. 2015;36(7):709–715. doi: 10.1016/j.placenta.2015.04.006.

8. Kim SY, England L, Wilson HG, C. Bish, Satten GA, et al. Percentage of gestational diabetes mellitus attributable to overweight and obesity. Am J Public Health. 2010;100(6):1047–1052. doi.org/10.2105/AJPH.2009.172890

9. ACOG Practice Bulletin No. 190: Gestational Diabetes Mellitus. Obstet Gynecol. 2018;131(2):e49–e64. doi.org/10.1097/aog.0000000000002501.

10. Chiefari E, Arcidiacono B, Foti D, Brunetti A. Gestational diabetes mellitus: an updated overview. J Endocrinol Invest. 2017;40(9):899–909. doi.org/10.1007/s40618-016-0607-5.

11. Behboudi-Gandevani S, Amiri M, Yarandi RB,Tehrani FR . The impact of diagnostic criteria for gestational diabetes on its prevalence: a systematic review and meta-analysis. Diabetol Metab Syndr. 2019;11:11. doi.org/10.1186/s13098-019-0406-1

12. Metzger BE. International Association of Diabetes and Pregnancy Study Groups Consensus Panel. International association of diabetes and pregnancy study groups recommendations on the diagnosis and classification of hyperglycemia in pregnancy. Diabetes Care. 2010;33(3):676–682. doi: 10.2337/dc09-1848

13. The HAPO Study Cooperative Research Group. Hyperglycemia and adverse pregnancy outcomes. N Engl J Med. 2008;358(19):1991–2002. dx.doi.org/10.2337%2Fdb08-1112.

14. National Institute for Health and Care Excellence [Internet]. National Institute for Health and Care Excellence (2015): Diabetes in pregnancy: management from preconception to the postnatal period (NG3). 2015 [published 2015 Feb 25]. Available from: https://www.nice.org.uk/guidance/ng3.

15. Feig DS, Berger H, Donovan L, Godbout A, Kader T, Keely E, et al. Clinical Practice Guidelines Diabetes and Pregnancy Diabetes Canada Clinical Practice Guidelines Expert Committee. Can J Diabetes. 2018;42:S255–S282.doi.org/10.1016/j.jcjd.2017.10.038

16. American Diabetes Association. 2. Classification and Diagnosis of Diabetes: Standards of Medical Care in Diabetes-2020. Diabetes Care. 2020;43(Suppl 1):S14–S31.doi.org/10.2337/dc20-S002.

17. American Diabetes Association. 14. Management of Diabetes in Pregnancy: Standards of Medical Care in Diabetes-2020. Diabetes Care. 2020;43(Suppl 1):S183–S192. doi.org/10.2337/dc20-S014

18. Tan E, Scott EM. Circadian rhythms, insulin action, and glucose homeostasis. Curr Opin Clin Nutr Metab Care. 2014;17(4):343–348. doi.org/10.1097/mco.0000000000000061.

19. Catalano PM, Huston L, Amini SB, .Kalhan SC. Longitudinal changes in glucose metabolism during pregnancy in obese women with normal glucose tolerance and gestational diabetes mellitus. Am J Obstet Gynecol. 1999;180(4):903–916. doi: 10.1016/s0002-9378(99)70662-9.

20. Polsky S, Garcetti R. CGM, Pregnancy, and Remote Monitoring. Diabetes Technol Ther. 2017;19(S3):S49–S59. doi.org/10.1089/dia.2017.0023.

21. Scott EM, Feig DS, Murphy HR, Law G. Continuous Glucose Monitoring in Pregnancy: Importance of Analyzing Temporal Profiles to Understand Clinical Outcomes. Diabetes Care. 2020;43(6):1178–1184.doi.org/10.2337/dc19-2527

22. Wei Q, Sun Z, Yang Y, YU H, Ding H, Wang. Effect of a CGMS and SMBG on Maternal and Neonatal Outcomes in Gestational Diabetes Mellitus: a Randomized Controlled Trial. Sci Rep. 2016 Jan 27;6:19920. doi 10.1038/srep1992023.

23. McLachlan K, Jenkins A, O'Neal D. The role of continuous glucose monitoring in clinical decision-making in diabetes in pregnancy. Aust N Z J Obstet Gynaecol. 2007;47(3):186–90. doi: 10.1111/j.1479-828X.2007.00716.x

24. Battelino T, Danne T, Bergenstal RM, Amiel SA, Beck R, Biester T, et al. Clinical Targets or Continuous Glucose Monitoring Data Interpretation: Recommendations From the International Consensus on Time in Range. Diabetes Care. 2019;42(8):1593–1603. doi.org/10.2337/dci19-0028

25. Yogev Y, Ben-Haroush A, Chen R, Rosenn B, Hod M, Langer O. Diurnal glycemic profile in obese and normal weight nondiabetic pregnant women. Am J Obstet Gynecol. 2004;191(3):949–953. doi: 10.1016/j.ajog.2004.06.059

26. Harmon KA, Gerard L, Jensen DR, Kealey E, Hernandez T, Reece M, et al. Continuous glucose profiles in obese and normal-weight pregnant women on a controlled diet: metabolic determinants of fetal growth. Diabetes Care. 2011;34(10):2198–2204. doi: 10.2337/dc11-0723

27. Gaudet L, Ferraro ZM, Wen SW, Walker M. Maternal obesity and occurrence of fetal macrosomia: a systematic review and meta-analysis. Biomed Res Int. 2014;2014:640291. | doi.org/10.1155/2014/640291

28. Dutton H, Borengasser SJ, Gaudet LM, Barbour L, Keely E. Obesity in Pregnancy: Optimizing Outcomes for Mom and Baby. Med Clin North Am. 2018;102(1):87–106. doi: 10.1016/j.mcna.2017.08.008

29. Gibson KS, Waters TP, Catalano PM. Maternal weight gain in women who develop gestational diabetes mellitus. Obstet Gynecol. 2012;119(3):560–565.doi: 10.1097/AOG.0b013e31824758e0

30. Gomes D, von Kries R, Delius M,Mansmann U, Nast M, Stubert M et al. Late-pregnancy dysglycemia in obese pregnancies after negative testing for gestational diabetes and risk of future childhood overweight: An interim analysis from a longitudinal mother-

child cohort study. PLoS Med. 2018;15(10):e1002681. doi.org/10.1371/journal.pmed.1002681

31. Calabuig-Navarro V, Haghiac M, Minium J, Glazebrook P, Ranasinghe G, Hoppel C, et al. Effect of Maternal Obesity on Placental Lipid Metabolism. Endocrinology. 2017;158(8):2543–2555. doi.org/10.1210/en.2017-00152

32. Barbour LA, Hernandez TL. Maternal Lipids and Fetal Overgrowth: Making Fat from Fat. Clin Ther. 2018;40(10):1638–1647. doi: 10.1016/j.clinthera.2018.08.007

Table 1. Maternal characteristics of the studied patients

	CG (n=10)	OG (n=10)	p-value
Age (years).	33.50 [28.75–36.00]	32.0 [26.0–34.5]	0.5
Parity ≥ 1(n)	9	10	1.0
Pregestational BMI (kg/m^2)	22.15 [21.70–23.82]	39.95 [35.85–41.88]	<0.001
Weight gain (kg)	2.65 [0.00–8.57]	8.00 [5.50–10.75]	0.09
Family history of CVD (%)	30	20	1.00
Family history of diabetes (%)	40	50	1.00
Length of pregnancy (weeks)*	25.0 [24.0–25.0]	25.5 [24.0–28.0]	0.6
OGTT (mg/dL)			
Fasting	75.50 [72.00–79.75]	81.50 [74.25–87.00]	0.08
1 hour	129.0 [117.0–141.0]	134.0 [120.0–161.0]	0.4
2 hours	110.00 [95.25–116.00]	109.00 [93.75–124.50]	0.9

* Length of pregnancy at the time of oral glucose tolerance test,OG: obese group, CG: control group, BMI: body mass index; OGTT: oral glucose tolerance test; wk: week; CVD: cardiovascular disease. Data are medians, Interquartile range (IQR), and percentage. P-values are calculated using by Wilcoxon-Mann-Whitney test and chi-squared test.

Table 2. Continuous glucose monitoring data in control and obese groups

	CG	OG	p-value*
**Glucose (mg/dL)**			
Before breakfast	77.77 ± 10.55	82.02 ± 11.06	<0.01
1 hour after breakfast	94.25 ± 15.70	97.26 ± 11.06	0.8
2 hours after breakfast	87.31 ± 13.10	93.48 ± 18.74	<0.001
Before lunch	82.77 ± 15.15	85.26 ± 15.65	0.2
1 hour after lunch	97.74 ± 13.60	97.71 ± 14.96	0.6
2 hours after lunch	93.78 ± 12.30	91.13 ± 13.65	0.15
Before dinner	82.80 ± 2.75	86.68 ± 2.04	0.08
1 hour after dinner	94.42 ± 19.05	94.02 ± 17.35	0.8
2 hours after dinner	90.65 ± 23.37	92.78 ± 20.27	0.2
Daytime	87.58 ± 15.40	93.08 ± 18.30	<0.001
Nighttime	79.35 ± 15.76	84.73 ± 16.31	<0.001
**AUC (mg/min/dL)**			
Day	65.56 ± 0.144	67.47 ± 0.105	<0.001
Night	19.53 ± 0.072	20.42 ± 0.05	<0.001
24 hours	85.08 ± 0.161	87.89 ± 0.116	<0.001

CG: control group, OG: obese group, AUC: area under the curve. Preprandial and postprandial glucose level is the mean of three consecutive values before or after the respective meal. Daytime glucose is the mean glucose level between 6:00 am and 12:00 pm. Nighttime glucose is the mean glucose level between 12:00 pm and 6:00 am. Daytime AUC is between 6:00 am and 12:00 pm and nighttime AUC is between 12:00 pm and 6:00 am.*The p-values (obese vs. control) are based on F statistics for comparisons test.

Table 3. The Mixed Linear Model to analyze the effect of obesity on the glucose levels.

	**CG (95.0% CI)**	**OG (95.0% CI)**	**p-value**
Whole sample	84.94 (81.55; 88.33)	88.58 (85.43; 91.63)	0.17
Daytime	86.87 (82.90; 90.84)	90.21 (87.20; 93.24)	0.25
Nighttime	78.10 (72.61; 83.60)	82.78 (78.60; 86.96)	<0.001

 Click here to access/download;Figure;22.tif

Figure 1. Glucose profile in pregnant women with normal glucose tolerance

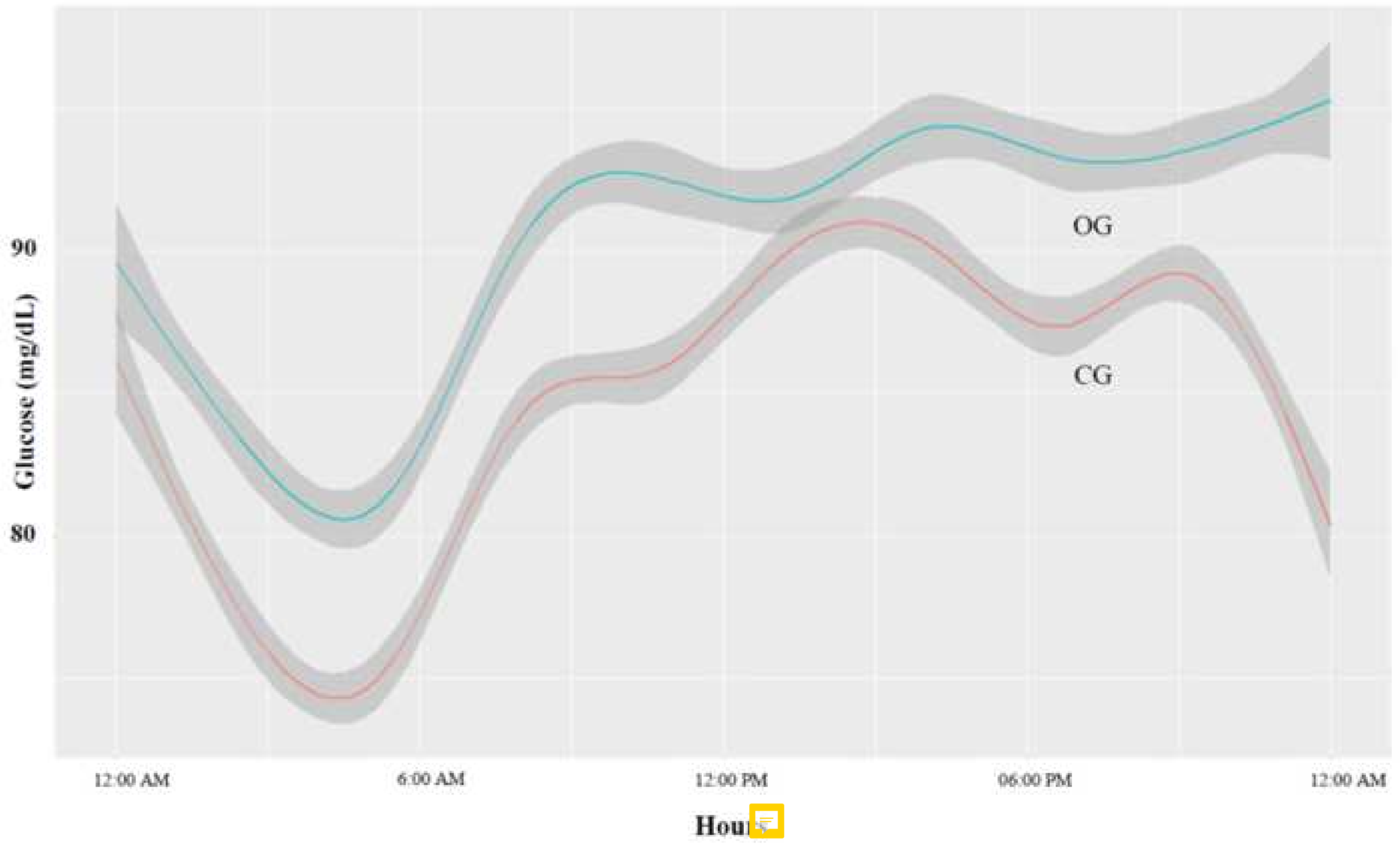

Obese group (n=10) is represented by the green smooth curve (lambda=1,000,000) and Control group (n=10) by the red smooth curve.

Click here to access/download
Supporting Information
renamed_20b2f.docx
