# Supplementary material for: Continuous glucose monitoring in obese pregnant women with no hyperglycemia on glucose tolerance test
Source: PLoS One. 2021 Jun 10;16(6):e0253047. doi: 10.1371/journal.pone.0253047 (PMC8191902; doi:10.1371/journal.pone.0253047)
Supplement: Attachment — Submitted filename: PONE-D-20-32574_reviewer.docx [file pone.0253047.s001.docx]

**PLOS ONE**

**Manuscript No.** PONE-D-20-32574                                                         03^th^ December, 2020

 Dear Editor Muhammad Sajid Hamid Akash, PhD

**TITLE****:**   **Continuous glucose monitoring in obese pregnant women with no hyperglycemia on glucose tolerance test**

**Author:** Rosa Maria Rahmi.

**Comments to the Author**

This manuscript nice presented the interesting finding that study the continuous glucose monitoring performed in obese pregnant women with normal oral glucose tolerance test with 75 g of glucose between the 24th and the 28^th^ gestational weeks*.* Study was nicely designed to compare 24-hour glycemic levels between obese pregnant women with normal glucose tolerance and non-obese pregnant women. Overall, these findings are important and interesting. However, further improvement is necessary to solidify the Manuscript.

Here are a few comments and questions:

1. In current study author compare the obese (OG) And the (CG) between the 24th and the 28th gestational weeks. But every woman gain weight during pregnancy period. How Author compare between obese and control?
2. Pregnancy of each women i.e. 1^st^ pregnancy, 2^nd^ pregnancy or 3^rd^ pregnancy data?
3. Diet plan absent in current study. Diet plan morning, lunch, dinner. Some food readily absorbed; Spicy food take more time to absorbed?
4. Time duration between breakfast, lunch and Dinner not mentioned?
5. There are some grammar errors. revise the manuscript.

Authors address these deficiencies, then the manuscript should be considered for publication.
